# The role of chest ultrasonography in the management of respiratory diseases: document I

**DOI:** 10.1186/2049-6958-8-54

**Published:** 2013-08-09

**Authors:** Alessandro Zanforlin, Rosangela Giannuzzi, Stefano Nardini, Americo Testa, Gino Soldati, Roberto Copetti, Giampietro Marchetti, Salvatore Valente, Riccardo Inchingolo, Andrea Smargiassi

**Affiliations:** 1 Pulmonary Medicine Unit, GeneralHospital, Trecenta, Rovigo, Italy; 2 Emergency Department, UniversitàCattolica del SacroCuore, University Hospital “A. Gemelli”, Roma, Italy; 3 Pulmonary and TB Unit, VittorioVeneto GeneralHospital, Vittorio Veneto (TV), Italy; 4 Internal Medicine Unit, Private Hospital “Madonna delle Grazie”, Velletri, Roma, Italy; 5 Emergency Department, General Hospital “ASL 2 Valle del Serchio”, Castelnuovo di Garfagnana, Lucca, Italy; 6 Emergency Department, GeneralHospital “ASS 5 BassaFriulana”, Latisana, Udine, Italy; 7 Pulmonary Medicine Department, “SpedaliCivili”, Brescia, Italy; 8 Pulmonary Medicine Department, UniversitàCattolica del SacroCuore, University Hospital “A. Gemelli”, Roma, Italy

**Keywords:** Chest ultrasonography, Diaphragm, Pleural diseases, Pneumothorax

## Abstract

Chest ultrasonography can be a useful diagnostic tool for respiratory physicians. It can be used to complete and widen the general objective examination also in emergency situations, at the patient’s bedside. The aim of this document is to promote better knowledge and more widespread use of thoracic ultrasound among respiratory physicians in Italy. This document I is focused on basic knowledge of chest ultrasonography technique, physical basis, aims and characteristics, fields of application. Document I shows how chest ultrasonography can be useful to detect and monitor pleural diseases, pleural effusions and pneumothorax and how it can assess diaphragmatic kinetics and pathologies.

## Review

### Introduction

Ultrasonography is currently underutilized as an imaging method in the respiratory field in Italy and this despite the fact that it is an easy-to-learn method with simple, straightforward signs. In reality, given its practicality and noninvasive nature, it can be used to complete and widen the general objective examination so as to facilitate a rapid diagnosis also in emergency situations, at the patient’s bedside.

In spite of these characteristics, chest ultrasound tends to be more used in emergency departments and intensive care than in respiratory divisions.

The aim of this document is to promote a better knowledge and more widespread use of thoracic ultrasound among respiratory physicians in Italy; hence this document is addressed to all fellow specialists involved in the field of respiratory disease. The present document will serve also as a basis for specific training at theoretic level.

A following document (Document II) will deal with advanced approaches to chest ultrasound like in differential diagnosis of sonographic interstitial syndrome, differential diagnosis of cardiogenic and non-cardiogenic acute pulmonary edema, raising diagnostic suspicion of pulmonary embolism, ultrasound characterization of lung consolidations, ultrasound-guided interventional procedures, lung ultrasound in neonatal and pediatric care.

### Field of use

Although ultrasound can be used to explore multiple districts for different purposes (especially in emergency and intensive care) in the respiratory setting, the routine use of ultrasound is as a tool at the patient’s bedside to aid and confirm the diagnosis of diseases suspected following and integrating the objective examination; it can be completed by the common techniques of chest imaging; as well it can help guide eventual interventional procedures that the clinician may decide necessary [1].

The strong points of ultrasonography are as follows:

– The technique and its sonographic signs are simple to learn.

– It offers a practical tool to complete and expand the general objective examination, facilitating a rapid diagnosis even in critical conditions.

– It is useful as a “bedside” tool: for establishing the pharmacological treatment, monitoring its efficacy, and guiding eventual diagnostic/therapeutic interventional procedures.

In the clinical practice of respiratory specialists thoracic ultrasound can be used to investigate:

1. the chest wall

–musculoskeletal, subcutaneous structures, etc.

2. position, morphology and motility of the diaphragm

evaluation of diaphragmatic excursion and thickness and diagnostics of paresis, paralysis, hypomobility.

3. the pleura

assessment of pleural effusions: also minimal effusions not visible at standard X-ray can be identified and estimated volumetrically; detection of adherences and lobulations; characterization of the effusion (“corpusculated, non corpusculated, empyema”); secure guiding in diagnostic interventions (“thoracentesis, drainage, thoracoscopy”).

Identification of pneumothorax (PNX), also that which is occult at chest X-ray, especially in patients unable to maintain erect stance.

Maximum accuracy in discerning the opacities and obliterations of the costophrenic recesses observed at X-ray (and which cannot be further characterized unless by CT scan).

4. the lung parenchyma

Assessment of “echographic interstitial syndrome”.

Assessment of lung parenchymal consolidations adjacent to the pleural line with identification of air bronchogram, fluid bronchogram (“bronchi filled with mucus or other fluid in the context of pulmonary consolidation”), evidence of areas of necrosis/abscess, signs at contrast-enhanced ultrasound and their follow up.

Description and characterization of areas of atelectasis.

By integrating these assessments with data obtained from echocardiography of the heart and blood vessels, there are numerous clinical situations that can benefit from the use of ultrasound:

• Integration of the objective examination in unstable/critical patients:

– pleuropulmonary assessment (acute lung injury [ALI]/acute respiratory distress syndrome [ARDS]), severe community-acquired pneumonia (CAP), cardiogenic pulmonary edema.

– diagnostic suspicion of pulmonary embolism by an integrated US approach (lung, heart, vein).

– estimation of central hypovolemia by evaluation of the inferior vena cava and heart chambers filling [2].

– detection, assessment and management of pleural effusions (“thoracentesis/ultrasound-guided drainage”).

– diaphragm kinetics evaluation (in weaning of ventilator-dependent patients)

– assessment of pneumothorax, a leading cause of preventable death in trauma patients;

– confirmation of endotracheal tube placement after intubation using the US sliding sign [3].

• Pleural disease investigation in “stable” patients:

– high risk of neoplasia

– interstitial lung diseases

– pregnant women (screening for pleuropneumonia)

– guide in performing pleural procedures (“ultrasound-guided

– thoracentesis/drainage/thoracoscopy”)

• System for monitoring/assessment of response to therapy:

– evolution of thickening/edema/pleural effusions.

– aid in PEEP titration in edematous states (cardiogenic vs. ALI/ARDS).

– titration of inotropic support and/or diuretics (qualitative/quantitative evaluation of cardiac contractile function in patients in shock).

### Basic knowledge

#### ***Physical basis of the ultrasonographic method***

Ultrasonography is based on the principle of the emission of ultrasounds and analysis of the echo thus generated. The ultrasounds utilizable in clinical practice have frequencies ranging between 2.5 and 20 MHz. In the respiratory setting, for an integrated sonographic examination, it is preferable to use convex probes (3–5 MHz) to examine the lung-pleura interface, pleural effusions, pleural thickening and costophrenic recesses; linear probes (7–12 MHz) for a more detailed study of the pleuro-parenchymal interface and of the chest wall; and sector probes to integrate data from the echocardiography. Higher frequencies, using linear probes, allow to get more details in terms of higher axial resolution power but they cannot permit the evaluation of deeper structures. Linear probes are useful to superficial and detailed studies. Convex probes are useful to assess deeper structures without high axial resolution power [1].

The probe is placed lightly on the skin of the body area being tested, which has been prior spread with a layer of ultrasound gel to eliminate any air that may be eventually present (air, like any other gas, and bony structures are barriers to ultrasound waves, creating interfaces with high acoustic impedance). The echo (reflection) is generated by the difference in the acoustic impedance which in turn is caused by the different composition of the structures invested by the sound wave. This echo is picked up by the probe itself.

The signal picked up by the probe is then elaborated by a calculator and converted into a two-dimensional image on a screen.

#### ***Basic knowledge about the ultrasound device, probes and their modes of use***

There exist substantially three types of ultrasound device that provide different services while differing in terms of their portability, which is linked in turn to their weight and size: 1) “hand-held” devices, the smallest and most convenient to handle, are a sort of extension of the stethoscope that fit in the pocket; 2) medium-sized portable ultrasound devices that are suitable for use in the hospital ward; 3) finally, there are the large “installed” devices with which Diagnostic Imaging services are equipped, that are characteristically those of largest size and weight.

Ultrasonic testing can be carried out in “B-mode” (modulation of the brightness, that creates a bright dot for each reflected echo): it is the mode most frequently used in clinical practice producing images that can be interpreted in the light of current knowledge about normal and pathologic human anatomy. Ultrasound devices can also provide images obtained in “M-mode”, which simply visualizes the movement of the body structures positioned along a line in the ultrasound beam over time. The representation of movement is obtained by the quick succession of sound impulses emitted.

Ultrasound devices are generally equipped with a knob or key to allow adjustment for depth and to amplify the return echo (the sound band weakens as it penetrates in depth and needs to be amplified to visualize better the structures that are most distant).

Conventionally, testing begins with the probe placed longitudinally on the parasternal line anteriorly (preferably in patients in supine position) or on the posterior axillary line/paravertebral line (preferably in patients lying sitting) - in this way the cranial end of the body structure examined will appear conventionally on the left of the screen and the caudal end on the right. Moving laterally from the parasternal and posterior-axillary/paravertebral position one can investigate the remaining lung fields in apical, mid-, and basal view. In this view, by locating the “curtain sign” on the parenchymal abdominal organs (liver, spleen, kidney etc.) one can make a good evaluation of the costophrenic recesses.

#### ***Basic knowledge about how the ultrasound image is formed and about normal and pathologic sonographic anatomy***

Alveoli are air bubbles distributed homogeneously in the interstitial “liquid”. A healthy lung is a wall of air that reflects the ultrasound beam because of its acoustic impedance. A probe placed in contact with the chest wall over a healthy lung reveals the pleural line, a hyper-reflective line lying beyond the tissues of the chest wall that constitutes the interface between body tissue and air. It is not possible to obtain real images of what lies beyond this line. Lung ultrasonography isn’t a morphological study but it allows only dynamic estimates and analyses of artifacts.

All structures that have a liquid content (e.g. pleural effusions) or are solid (e.g. parenchymal consolidations) or that, generally speaking, have an acoustic impedance similar to that of not-aerated tissues allow the ultrasound beam to pass through them and not only can they be well visualized but they permit also the visualization of adjacent structures [1].

The first structure investigated in a normal lung is the so-called pleural line, generated in the interface between chest wall and aerated lung. It moves with the act of breathing in and out (the image is defined as the “sliding lung” sign) [1]. The better way to study the pleural line is to use linear probes with high axial resolution power and avoiding harmonic images. If the subcutaneous tissue is very thick (e.g. obesity), using the convex probe is preferable.

Normal aerated lung shows the so-called A lines, i.e. artifacts that appear as parallel, equidistant lines below the pleural line (horizontal artifacts), and which represent reverberations of the acoustic interfaces situated above (“mirror effect”).

The so-called B lines (vertical artifacts) are also artifacts that project in depth originating from the pleural line and they are a marker of alterations of the very initial pleuroparenchymal layers that can modify the perfect “echo-reflecting” characteristics of the pleural acoustic interface (increase of the water/tissue content of the lung parenchyma with change of air spaces geometry in the initial subpleural layer of the lung parenchyma). In normal lung they are present only in limited number, if at all [4]. Vertical artifacts are described in document II.

#### ***Basic knowledge about the limits of ultrasound images, image interpretation, and the value of their clinical application***

The principal limit of lung ultrasound is the presence of subcutaneous emphysema that by definition impedes the penetration in depth of the ultrasound beam; other factors such as obesity, presence of chest wall hematomas or well developed musculature can create varying degrees of obstacle but they never impede the study of the lung.

Consolidated (“hepatized”) lung is clear to visualize thanks to the good sound band conductivity and it enables the carrying out of biopsies (see Document II).

The presence of a certain number of B lines is a signal of echographic interstitial syndrome and it can be assumed as an indication of cardiogenic or noncardiogenic pulmonary edema, infiltrative lung disease, or focal interstitial disease (see Document II).

‘Free-flowing’ pleural effusion (or else ‘loculated’ but adherent to structures that are good conductors of ultrasound - upper diaphragmatic effusion, in contact with the wall) is clearly visible at ultrasound as an image without echoes or weakly echogenic. The presence of pleural liquid allows the visualization of the lung parenchymal structures and of the other chest structures provided they are in contact with the effusion (e.g. heart, pericardium, diaphragm, mediastinal structures and vessels). In general in free-flowing pleural effusions the lower part of the lung, collapsed and rendered clearly visible, moves in syntony with the act of breathing. The density of the liquid can be estimated from the presence of liquid more or less corpusculated (transudate, exudate, hemothorax). The complexity of the effusion can be well estimated based on irregular hyperechogenic lines evidenced in the context of the effusion. Lines that modify their form following the act of breathing or heart beat are an indication of partial organization.

Lung ultrasound – providing it is carried out at the patient’s bedside – for the above-mentioned characteristics lends itself optimally as a guide to thoracentesis and other pleural procedures (see Document II).

Since the presence of a pleural line that moves with the act of breathing is indicative of lung “normally adhering to the parietal pleura”, its absence can allow to confirm the suspicion of pneumothorax.

In particular, this is true for the follow up of interventional procedures.

The diaphragm can be examined both by investigating the so-called “zones of apposition”, in which the diaphragm is anchored to the ribs, through the use of coronal scans with a linear probe, and by means of an ascendant subcostal approach with a convex probe, using the liver window on the right and the less useful spleen window on the left.

### Sonographic technique

Conventionally lung sonography is performed with the patient in a sitting position taking longitudinal scans starting anteriorly from the parasternal zone and posteriorly from the paravertebral/posterior axillary zones. In these scans, as one penetrates in depth from the surface one can visualize the skin and hypodermis, the pectoral muscles, 1 or 2 ribs according to their short axis, the intercostal muscles and the pleural line, at a deeper level with respect to that of the ribs, as a hyperechogenic line that moves [1].

From the parasternal and/or paravertebral position the examination will proceed as for auscultation with the stethoscope, moving laterally up and down until the costophrenic recess is reached. At this level, both on the right and on the left, one can visualize the so-called “curtain sign”, the caudal extreme of both lungs, at the moment in which it involves the respective costophrenic recess during the act of inspiration, “covering” abdominal structures like the hepatic parenchyma on the right and the spleen parenchyma on the left as though it were pulling the drapes of a curtain over them. Curtain sign represents the boundary between “morphological” and “artifactual” ultrasonography.

Below the pleural line, the normally aerated lung appears “black”; there can be present the above-mentioned A lines, horizontal reverberations without any pathologic implication, and sometimes a few vertical artifacts which, if limited in number, do not indicate any pathology.

Additional scans that allow to better characterize and investigate eventual lesions or pathologic alterations are the transverse or, better still, intercostal scans [5].

### Pleural disease

In normal conditions ultrasound does not physically show us the pleura, visceral and parietal, but evidences the solid/air interface that we describe as the “pleural line”. A pathologic involvement of the pleura or of the pleural space can however “double” the line, making it possible to distinguish the two pleurae. The pathologic aspects of the pleura that can be investigated by ultrasound are: effusions, pneumothorax and pleural thickening.

### Pleural effusions

The identification of a pleural effusion can be carried out with the probe in a cranio-caudal direction and proceeding with longitudinal scans from the apex down to the base of the lung. Fluid of whatever nature (inflammatory, transudative, hematic, etc.) that accumulates in the pleural space causes a separation of the parietal and visceral layers of the pleura, appearing as a prevalently non-echogenic area that collects between the lung parenchyma and the chest wall. At the base of the lungs the curtain sign is no longer present due to the accumulation of fluid in the bottommost parts of the chest, while it is possible to see the dome of the diaphragm, also in this case appearing as a hyperechogenic interface surrounding the liver; with a higher-frequency linear probe the diaphragm can occasionally be seen as a muscle, i.e. as a hypo-echogenic echo structure.

**Figure 1:**
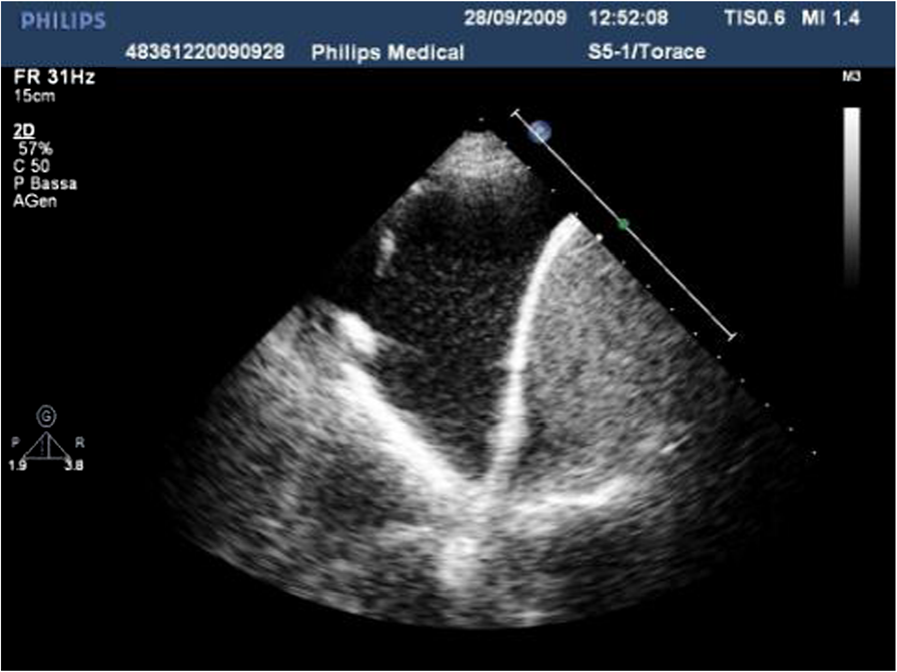
Posterior longitudinal scan in a seated patient, with sector probe showing a free-flowing pleural effusion at the base of the hemithorax.

Fluid accumulations of small dimension can be investigated with the linear probe, but for a complete examination of most effusions an in-depth study is necessary, possible only with a low-frequency probe (convex or sector) (Figure 1) [6].

A pleural effusion must be characterized in terms of its localization, quantity, its sonographic aspect and eventual collateral findings.

#### ***Localization***

Effusions are defined as free-flowing when they accumulate by gravity and their position varies with change to decubitus position.

In free-flowing effusions, the pleural liquid involves initially the costophrenic recess; it thus forms at the base of the chest, between the diaphragm and base of the lung. As the volume increases it rises up the parietal pleural space and progressively compresses the lung parenchyma of the inferior lobe that fluctuates within the liquid and progressively loses its sonographic structure of artifact, assuming a “parenchymatous” aspect: within it one can in this phase detect zones of partial aeration composed of hyperechogenic areas. With further increases of the liquid and its pressure, the inferior lobe retracts to the hilum. When the effusion is massive it also compresses the superior lobe to the point of causing the collapse of the whole lung to the hilum [7].

**Figure 2:**
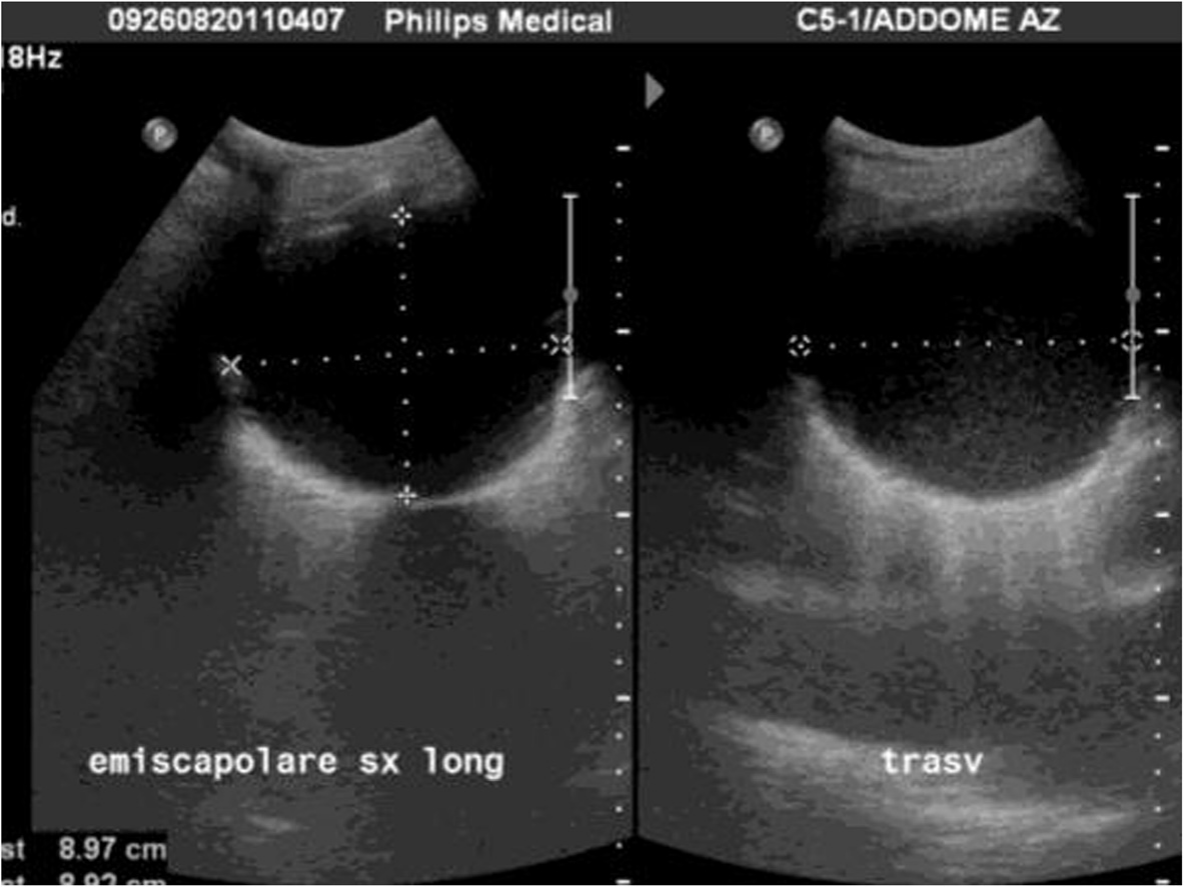
**Longitudinal and transverse scan with convex probe of a loculated effusion.** It is well demarcated and has a clear contour.

Effusions are defined as loculated when they are fixed and limited to a restricted area by a wall usually of a fibrinous nature or post-inflammatory pleural adherences; in this case the position is indicated using as coordinates the intercostal spaces and the vertical lines of the thorax (Figure 2).

#### ***Quantification***

While quantifying a loculated effusion can be simple, i.e. limited to measuring the tridimensional diameters particularly when circumscribed and completely accessible, ultrasound is not currently able to quantify with precision a free-flowing pleural effusion: the pleural cavity in fact has a complex geometry and is difficult to approach, making it difficult to devise formulas for calculation. At the moment, therefore, the only way to quantify the effusion is by visual estimate on the part of the operator. Alternatively, it is possible to measure the depth of the effusion or its longitudinal diameter, that often correlate with the quantity of liquid [8,9].

Nevertheless, the simple measurement of the quantity of liquid present in a pleural cavity may not be indicative of the real clinical implications of the effusion under examination since the same quantity of effusion can cause different signs and symptoms in patients of differing stature and constitution. Moreover, the descriptive quantification of the effusion is subjective, often presenting some inter- and intra-operator variability. A quantitative classification of free-flowing pleural effusion has been proposed based on the degree of involvement of the pulmonary lobes (where the presence of artifacts is a sign of aeration and thus of a “functioning” lobe) and on the presence of sonographic findings (diaphragmatic dome, presence of artifacts in the lobe, pulmonary hilum visible, intercostal spaces) in order to provide a measure which has inter-rater repeatability [7] (Table 1).

**Table 1:** Sonographic anatomic classification based on quantity of pleural effusions (in longitudinal scan from the posterior axillary line)

**Grading**	**Description**	**Landmarks**	**Intercostal spaces**
***Grade 1 Minimum***	Limited to costophrenic sinus	Diaphragmatic dome partially evident	Limited to costophrenic sinus
***Grade 2 Small***	Lower lung lobe partially involved	Diaphragmatic dome completely evident	1 intercostal spaces
***Grade 3 Small to Medium***	lower lung lobe partially collapsed	Lower lung lobe partially atelectasic. Pulmonary hilum not visible	2-3 intercostal spaces
***Grade 4 Medium***	Lower lung lobe completely collapsed	Atelectasis of the lower lung lobe. Pulmonary hilum visible	3-4 intercostal spaces
***Grade 5 Large***	Upper lung lobe partially involved	Atelectasis of the lower lung lobe. Upper lung lobe partially atelectasic.	4 intercostal spaces or more
***Grade 6 Massive***	Lung fully collapsed	Atelectasis of the whole lung. Hilum completely visible	

#### ***Echographic aspect***

Chest radiography, in particular if done in bed, has poor sensitivity for revealing pleural effusions [10], especially if it is not carried out in optimal conditions. In addition, it has difficulty evidencing correctly eventual compartments or loculations present within the effusion itself; these will appear on the X-ray as an opacity of non-univocal interpretation and thus will require further investigation to be diagnosed [11].

Computerized tomography (CT), instead, is very sensitive in identifying even tiny effusions and in fully characterizing them; nonetheless the notable quantity of radiation to which the patient is exposed, its high costs and the difficulty in carrying out a daily monitoring of the effusion due to the above-mentioned marked exposure to radiation renders its use unjustified and also not recommendable in this sense.

Ultrasound not only permits the evidencing of even small quantities of effusion, but it also allows their characteristics to be defined and, above all, it allows a daily monitoring of the effusion’s size without recourse to radiation and in a completely harmless mode for the patient. Moreover the use of portable ultrasound devices permits also greater comfort for the patient who can undergo examination at the bedside, without the need for inconvenient transfers.

The echographic aspect of pleural effusions varies according to the chemo-physical characteristics of the liquid and is generally classified into 4 sonographic patterns: 1) anechoic (few floating corpuscles), 2) complex non-septated (containing floating corpuscular material of variable density), 3) complex septated (composed of fibrin strands or septae in a lattice-like pattern), and 4) homogeneously hyperechogenic (densely corpusculated) [12].

**Figure 3:**
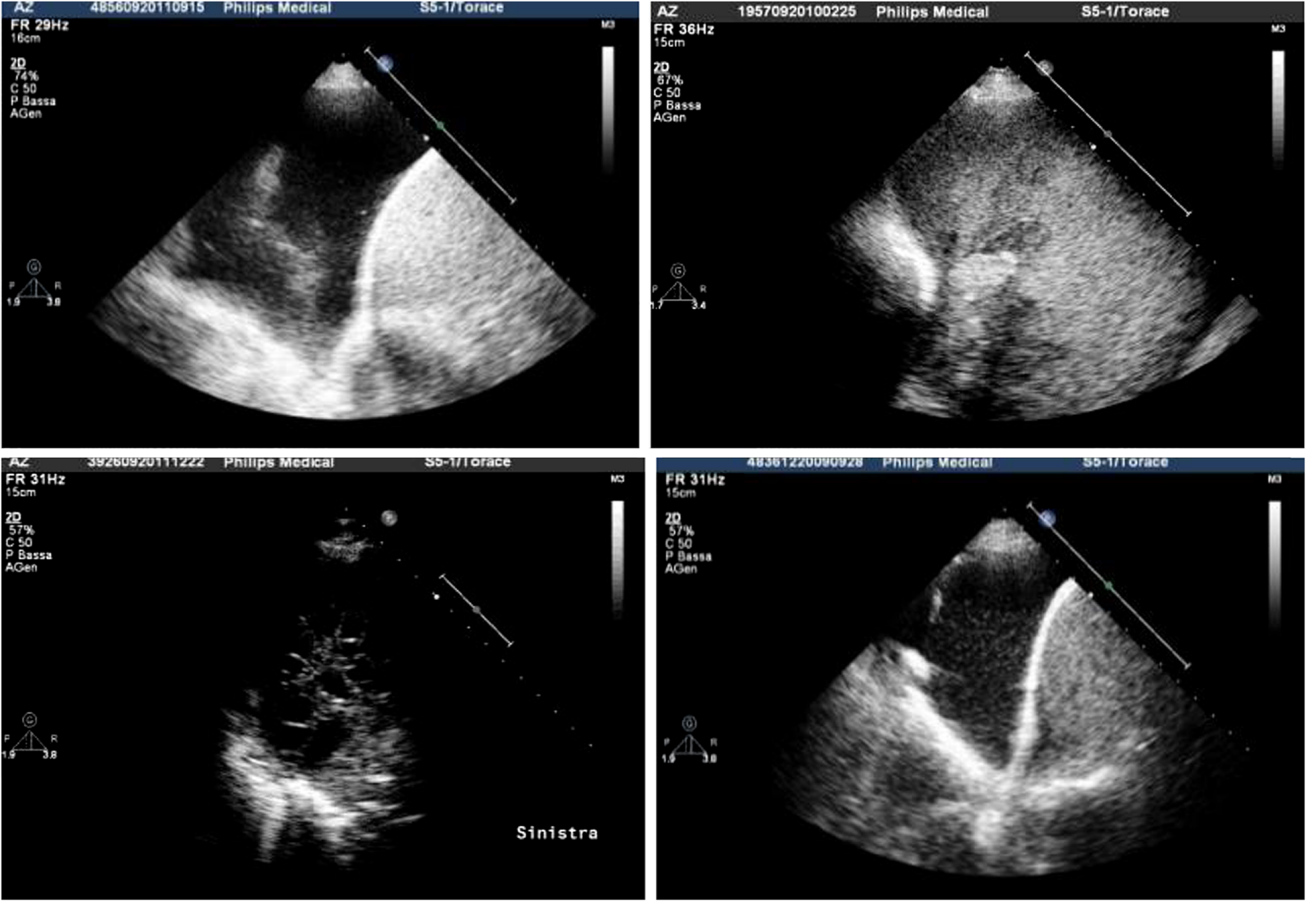
Upper left, anechoic effusion; upper right, echogenic effusion; lower left, complex septated effusion; lower right, complex non-septated effusion.

Thanks to ultrasonography, the early identification of an organized pleural effusion, empyema or hemothorax accelerates the diagnostic process making thus possible an earlier, more effective intervention (Figure 3).

#### ***Collateral findings***

**Figure 4:**
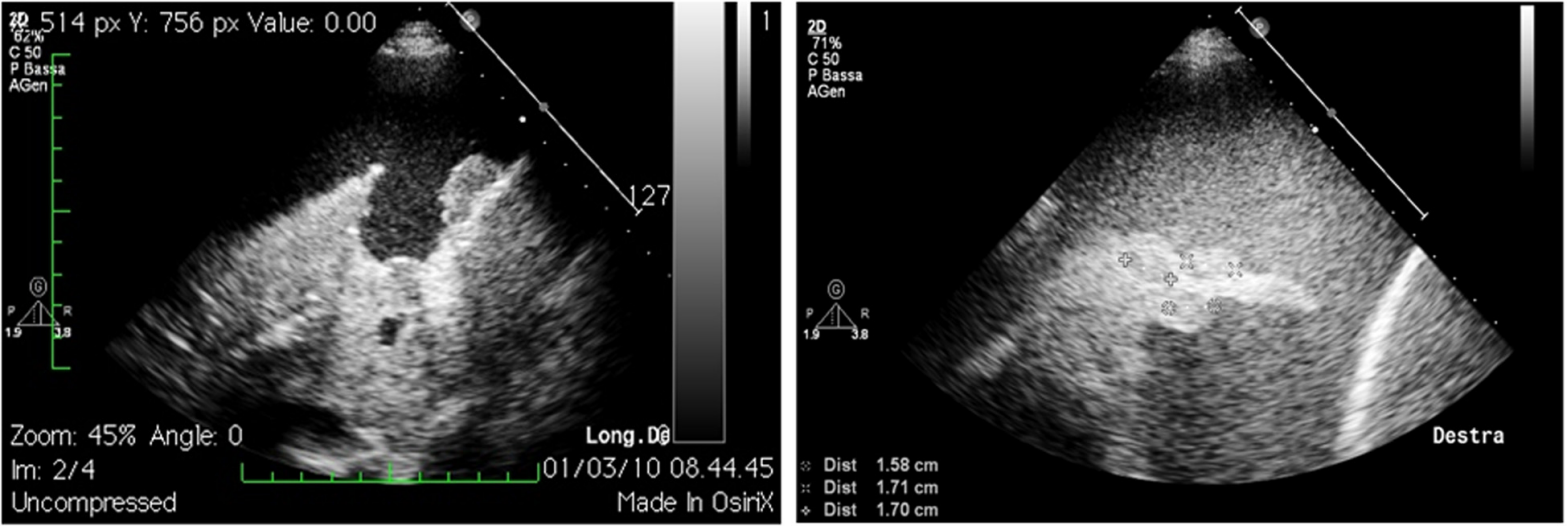
Collateral findings in the diagnosis of pleural effusion: on the left, nodulations of the diaphragmatic profile; on the right, hypoechogenic nodules in the collapsed lung parenchyma.

Pleural effusion renders the diaphragm and lung parenchyma visible, making it possible to describe eventual collateral findings that can provide useful indications about the nature of the effusion itself: on the diaphragm profile there may be present nodules or growths, indicative in general of neoplastic disease. From the collapsed lung parenchyma one can obtain information on its state of aeration (if hyperechogenic spots are present they indicate the presence of air), on perfusion (by echo Doppler testing with the correct inclination of the probe it is sometimes possible to see pulsing the main branches of the pulmonary circuit) or nodules can be “unmasked” within it (Figure 4).

### ***Pneumothorax***

Knowledge about artifacts of the normal lung is fundamental for identifying or excluding a pneumothorax.

The hyperechogenic line that constitutes the interface between chest wall and lung and which takes the name of “pleural line” is formed by the parietal pleura and by the visceral pleura that slides along the former during respiration. This movement, evident in sonography as a flicker, takes the name of “sliding sign”. It was noted for the first time by a veterinarian, Rantanen, who diagnosed in horse the presence of pneumothorax precisely by the absence of this sliding [13].

Ultrasound for the diagnosis of pneumothorax is highly sensitive particularly for the small hidden pneumothoraxes that classically remain invisible at traditional X-ray and, in the supine patient, are discovered in the so-called area of the “deep sulcus”, that radiographic sign consisting of a more pronounced hypodiaphany that surrounds the area of cardiac dullness and permits diagnosis of small pneumothoraxes. In supine decubitus position, air gathers in the zones where the downward inclination is least, that correspond precisely to the radiographic deep sulcus. Thus, an ultrasound examination for the diagnosis of pneumothorax should be performed with the patient in supine position, using linear or also convex probes in the lower right and left parasternal sites, moving more cranially or caudally depending on the anatomic conformation of the thorax [14].

The sonographic diagnosis of pneumothorax consists in: the absence of sliding sign, absence of B lines, these being vertical artifacts arising from the visceral pleura and thus not observable at the moment in which air is stratified between the two pleural layers [15], and the presence of “lung point(s)”, a pathognomonic and highly sensitive sonographic sign of pneumothorax. This is the point at which the two pleurae get back to adhere; at this point will be visible, in part, the sliding sign with eventual vertical artifacts and, in part, an immobile hyperechogenic line with horizontal artifacts below (A lines); it is in this half of the ultrasound scan that the pneumothorax appears [16,17]. In the pneumothorax, especially on the left, the transmission of cardiac systoles on the pleural line, the so-called “lung pulse”, is lost.

The last sonographic sign that is found in the case of pneumothorax, but which is not diagnostic in that it is present also in normal conditions or in presence of emphysematous bullae, is the so-called “A line pattern” [17,18] due to strong reverberations of the ultrasounds on the highly impeding interface created by air like in “hyper-mirror” effect.

The use of M-mode for the diagnosis of pneumothorax is a rather dated method and should be not routinely used; it is done by activating the M-mode in the zone in which sliding is not present, thereby obtaining a so-called “barcode” aspect (as opposed to the “seashore” sign found in zones where sliding is present) [15].

Ultrasound has demonstrated a superior sensitivity and similar specificity with respect to standard radiography in the diagnosis of pneumothorax, but it is not sufficient to quantify it, and to this end must be integrated with the other radiologic investigations, in particular CT [19].

### Pleural thickening

Ultrasound permits to evaluate thickening of the pleura [20], be it of an inflammatory or neoplastic nature, even if it is not possible to evaluate the totality of the pleural surfaces since the mediastinal pleura remains inaccessible as well as part of the parietal pleura, masked by the shoulder blades and armpit.

Echo-free thickening covering some areas of the lung like a “shirt” may seem at first sight to be pleural effusions; however, observing attentively the interface between the anechoic area and lung, one can note that there are irregularities in its profile that remain fixed to the chest wall and do not follow the sliding, making it seem as though the lung slides on a solid structure, identifiable thus as a pleural thickening.

This aspect is typical of fibrous thickening due to outcomes of chronic inflammatory processes. In the presence of a calcified pachypleuritis, the thickening can present irregular hyperechogenic areas (calcifications) that impede the view of the lung parenchyma generating shadow cone artifacts [21].

**Figure 5:**
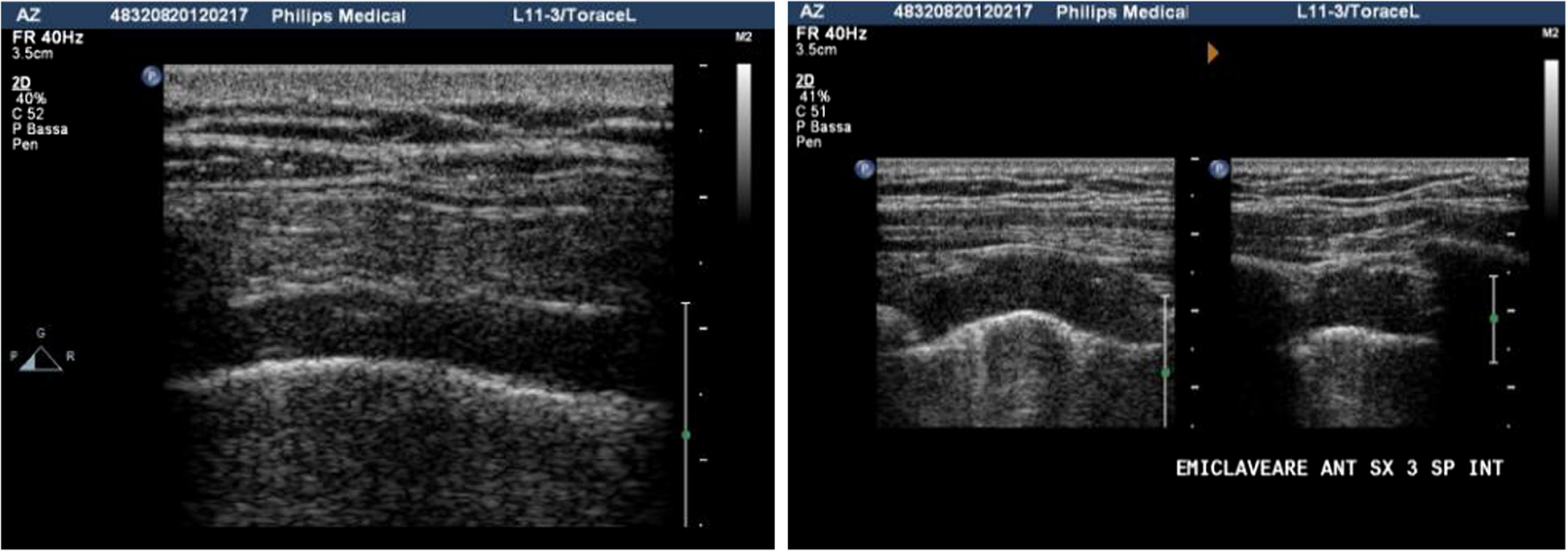
Left, pleural thickening caused by a plaque due to asbestosis: the hypo-anechoic area causes a doubling of the pleural line; right, pleural plaque observed in transverse and longitudinal scan that shows infiltration of the wall muscle layers, suggestive of neoplastic transformation.

Pleural plaques appear as well-demarcated areas of hypo-anechoic thickening in transverse and longitudinal scan; if they contain calcifications, these appear within the plaque as discontinuous hyperechogenic lines with shadow cone below. In asbestosis it is important to evaluate the relationship between the areas of pleural thickening and the chest wall: in general pleural thickening areas are delimited in the pleural space, but if they penetrate across the intercostal spaces invading the layers of the wall (one can note an interruption of the intercostal muscle bands) this is suggestive of a neoplastic nature that requires further confirmation with other methods [21] (Figure 5).

**Figure 6:**
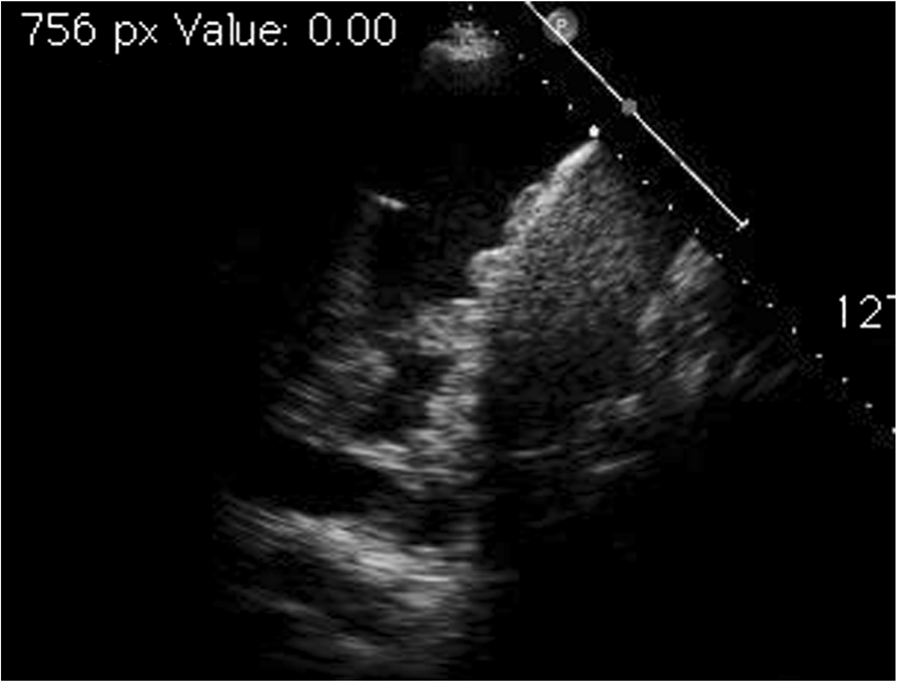
Growths of the diaphragmatic pleura observable in the presence of effusion, a sign of secondary involvement of the pleura.

The diaphragmatic pleura is well explorable in the presence of pleural effusions, when it is possible to observe hyperechogenic nodules or growths on the profile of the muscle. These findings, indicative of neoplastic pleural disease, are evidence of the malignant nature of the effusion (Figure 6).

### Sonographic evaluation of the diaphragm

The diaphragm is the principal muscle involved in respiration; paralysis and paresis of this muscle can result from abnormalities at any site along its neuromuscular axis, although they are most frequently due to diseases in the phrenic nerve or diseases of the muscle itself.

The techniques used for examining the diaphragm are either invasive or difficult, often requiring the use of radiation and consist chiefly in fluoroscopy, plethysmography, measurement of transdiaphragmatic pressures with a gastric-esophageal probe, or echo planar magnetic resonance (MR) imaging [22].

The use of B- and M-mode ultrasound to evaluate qualitatively diaphragm kinetics was introduced for the first time by Haber in 1975, evidencing the movement of the abdominal organs [23]. In the following years diverse approaches have been developed, longitudinal or transverse, with convex or linear probes, for the qualitative assessment of diaphragm kinetics both directly as well as indirectly (through the movement of abdominal structures such as the gallbladder, spleen or vena cava) [24].

**Figure 7:**
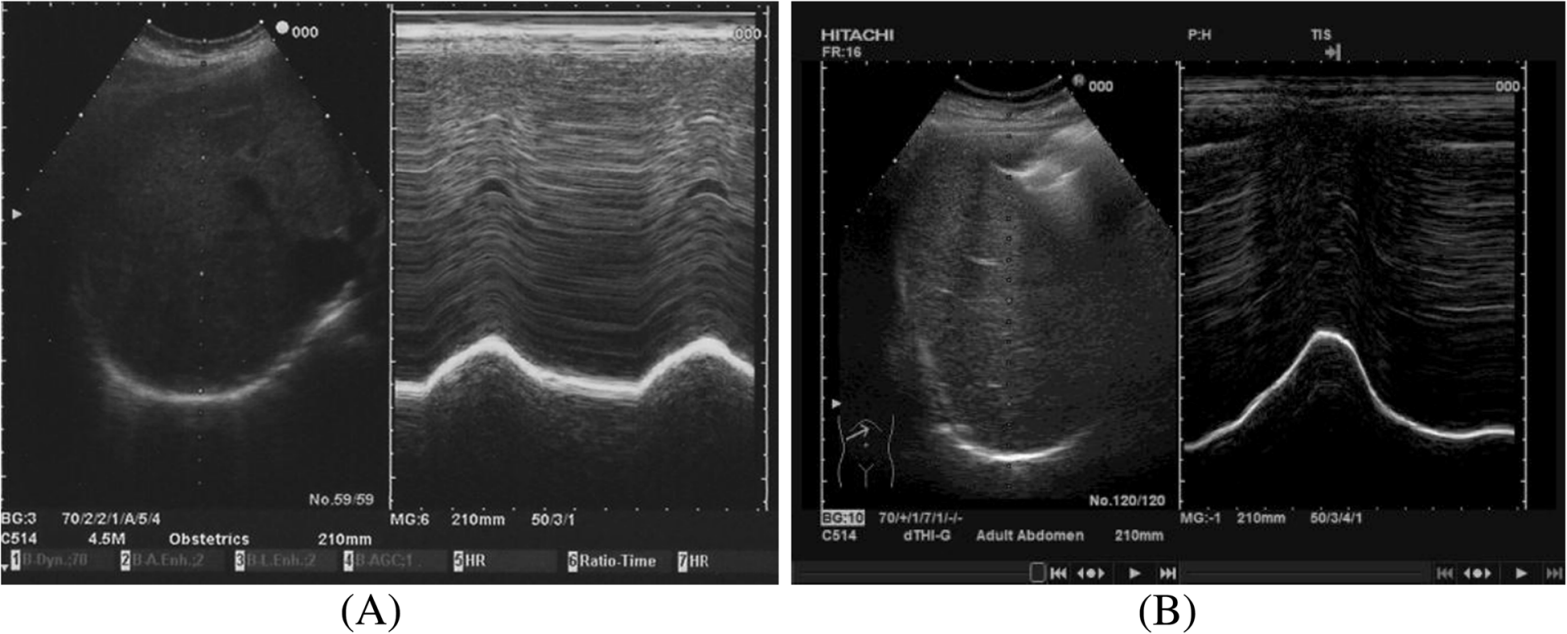
**Diaphragm in B- and M-mode in spontaneous breathing (A) and in forced respiration (B).**

**Figure 8:**
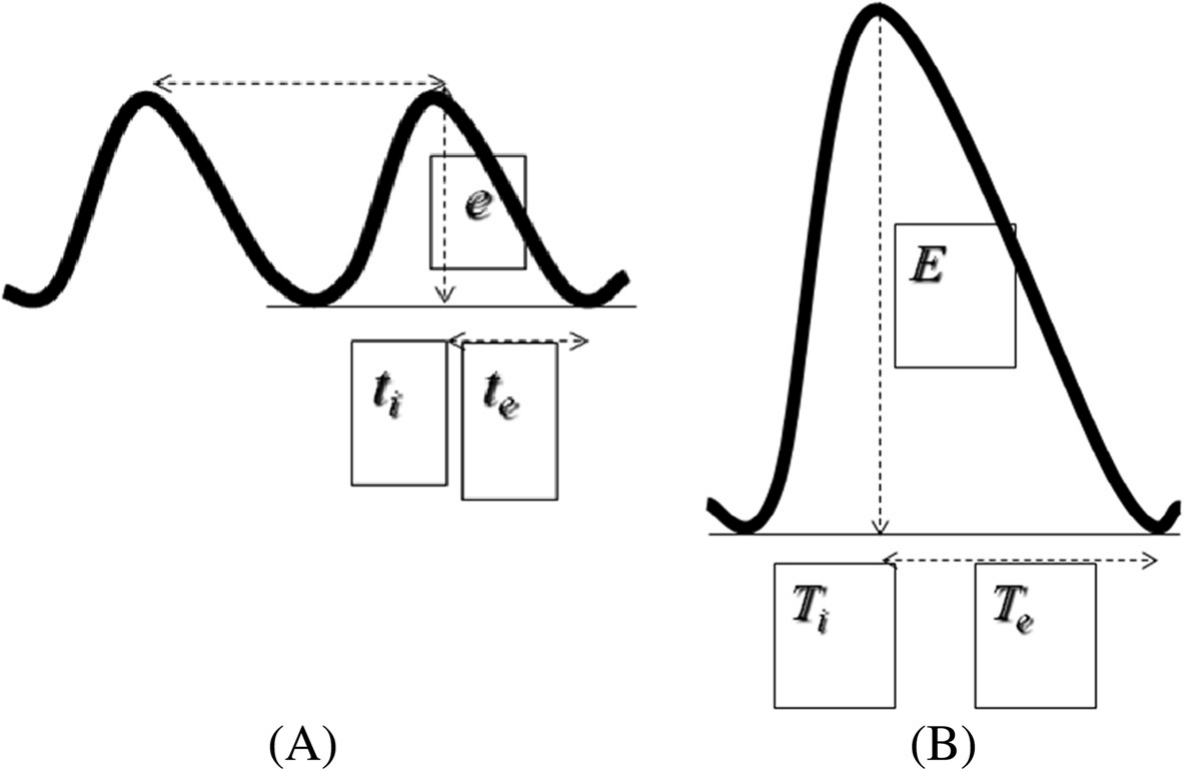
**Measure of the diaphragmatic excursion in spontaneous breathing (A), on average 1.8 cm.** Measure of the diaphragmatic excursion in forced respiration **(B)**, on average 7.8 cm. t_i_, inspiratory time; t_e_, expiratory time.

The approach we use is the anterior subcostal one using a convex probe. In B-mode the diaphragmatic interface appears as a hyperechogenic line surrounding the liver; at this point one tilts the probe to obtain the maximum convexity, using the gallbladder, where present, as a reference point. Inserting M-mode, one can visualize the movement of structures positioned along a line in the ultrasound beam. The diaphragmatic interface will appear in M-mode as a hyperechogenic line that assumes in time a sinusoidal form with the peak corresponding to maximum inspiration and the trough corresponding to expiration. On this M-mode trace one can perform measurements: the height of the curve corresponds to the diaphragmatic excursion which in spontaneous breathing is approximately 1.8 cm and in forced breathing reaches 7.8 cm [25] (Figures 7 and 8).

This method has proved simple and reproducible; however, among the limits linked to it are the difficulty represented by obese patients or patients suffering from intestinal meteorism and the difficulty at times in investigating the left hemidiaphragm which is complicated by the presence of air in the colon and by the smaller size of the spleen compared to the liver determining a smaller acoustic window.

Another method that investigates quantitatively the movement of the diaphragm is the measurement of muscle thickness, evaluated with linear probe in longitudinal scan at the level of the 8^th^-9^th^ intercostal space on the midaxillary line; these are the so-called zones of apposition where the diaphragm is apposed to the rib cage. During normal respiration diaphragm thickness is approximately 2.8 mm; during maximal inspiration the thickness reaches 4 mm or more and this appears to correlate with lung volumes [26].

## Conclusions

Chest ultrasonography can be a useful diagnostic tool for respiratory physicians to assess and monitor respiratory pathologies in many different conditions with wide field of application. This document I aims to focus on basic knowledge of chest ultrasonography, to describe sonographic assessment of pleural and diaphragm diseases and to promote more widespread use of this technique among respiratory physicians in Italy.

## Competing interests

The authors declare that they have no competing interests.
